# An analysis of the impact of Newcastle disease vaccination and husbandry practice on smallholder chicken productivity in Uganda

**DOI:** 10.1016/j.prevetmed.2020.104975

**Published:** 2020-04

**Authors:** Paul R Bessell, Roy Woolley, Stuart Stevenson, Lamyaa Al-Riyami, Patrick Opondo, Leslie Lai, Neil Gammon

**Affiliations:** a Epi Interventions. 17 Bangholm Avenue, Edinburgh, EH5 3AS, UK; b GALVmed, Doherty Building, Pentlands Science Park, Bush Loan, Penicuik, Edinburgh, EH26 0PZ, UK; c 3V Vets Franchise Limited, Tam Tam Opuke House, Akaidebe Cell, Dokolo Town Council, Dokolo District, Uganda; d Independent Consultant, Oakland, California, USA

**Keywords:** Chickens, Newcastle disease, Husbandry, Productivity, Vaccination, Smallholder

## Abstract

A number of studies have demonstrated the clear beneficial impact that vaccinating against Newcastle disease (ND) can have on reducing the frequency and severity of ND outbreaks. Here we go one step further and analyse the additional benefits in terms of improved production that result from vaccination.

Data were collected from a cross sectional survey in Uganda of 593 chicken-rearing smallholders (for the purpose of this study this was defined as a farm with fewer than 75 chickens). Consenting participants were administered a detailed questionnaire covering a range of aspects of chicken production and management. These data were subsequently analysed in a generalised linear model framework with negative binomial error structure and the total offtake over the previous 12 months (chicken sales + chicken consumption + chickens gifted) was included as the dependent variable. Different measures of flock size were tested as independent variables and the model was also offered the district of the flock, ND vaccine adoption, use of poultry housing, provision of supplementary feed and use of dewormers as potential independent variables. We also developed an analogous model for the offtake of eggs (sale and consumption).

The total size of the flock (counting chickens of all ages) was the measure of flock size that had the strongest association with offtake and was a significant but weak effect with an incidence rate ratio (IRR) of 1.011 (95 % Confidence intervals (CIs) = 1.007–1.015). ND vaccine adoption had a strong significant positive effect on offtake with an IRR of 1.571 (95 % CIs = 1.363–1.808). Use of a poultry house also had a significant effect (IRR = 1.365, 95 % CIs = 1.193–1.560). In the model of egg production, the number of hens was the demographic determinant with the lowest Akaike Information Criterion (AIC) (IRR = 1.094, 95 % CIs = 1.056–1.136) and ND vaccine adoption had a strong positive effect on egg offtake (IRR = 1.801, 95 % CIs = 1.343–2.412).

Vaccinating against ND has a clear beneficial impact on the productivity of the flock, and the livelihoods of smallholder farmers.

## Highlights

• An analysis of the impacts of Newcastle disease vaccination and husbandry practices on chicken flock productivity in smallholder farms in Uganda.
• Productivity was measured by offtake measured by the numbers of chickens consumed, gifted or sold over one year and eggs consumed and sold per week.
• · Adjusting for flock size, Newcastle disease vaccination resulted in a 57 % increase in offtake and use of poultry housing a 36 % increase in offtake.
• After adjusting for the number of hens, vaccinating against Newcastle disease resulted in an 80 % increase in egg production.
• Use of dewormers and feed supplementation had no significant effect.

## 1 Introduction

Smallholder poultry production is identified as a key stepping stone in the route out of poverty in developing countries on account of the rapid production cycles, low input requirements and the relative liquidity of poultry as an asset (Alders and Pym, 2009). Consequently, in developing countries, smallholder chicken production accounts for 70 % of chicken production (Alders and Pym, 2009). However, outbreaks of disease and in particular virulent Newcastle disease (ND) which is often associated with high mortality (up to 100 %), place a considerable constraint on the productivity of chicken flocks (Aboe et al., 2006; Otim et al., 2007; Harrison and Alders, 2010). Inexpensive vaccines that are effective and easy to administer (Tu et al., 1998; Alders and Spradbrow, 2001) are available and have been shown to be effective at reducing mortality rates among infected flocks (Harrison and Alders, 2010; Alders, 2014; Bessell et al., 2017). Consequently, strategies are being developed to improve rates of adoption of ND vaccines but in spite of this, adoption rates and compliance (in terms of frequency of vaccination) are variable (Alders, 2014; Bessell et al., 2017; Campbell et al., 2018).

ND vaccines have been shown to have a beneficial impact in terms of reducing disease incidence and enabling the production of larger flocks, albeit with larger flocks more likely to vaccinate (de Bruyn et al., 2017). However, further evidence quantifying the role of ND vaccination and other interventions in impacting flock productivity would provide key instruments for driving advocacy and understanding the benefits for adoption of ND vaccines both within countries with endemic virulent ND and from among international stakeholders. Small scale studies have addressed this in detail at fine scales (Jugessur et al., 2006; Njue et al., 2006; Henning et al., 2013), so here we propose to review changes in productivity resulting from ND vaccination where it has been promoted across large areas. We consider this over a 12 month period during which there will be peaks in demand for chicken meat (for example associated with religious festivals) and peaks in incidence of ND.

A further component that has been identified as a key step in improving flock productivity are improvements in poultry husbandry (Henning et al., 2009; Rodríguez et al., 2011; FAO, 2014). This includes the use of a poultry house offering protection from predators and from escape (Ahlers et al., 2009; Desta and Wakeyo, 2013; FAO, 2014), supplementary feeding to improve the weight and fertility of the chickens (Ahlers et al., 2009; Henning et al., 2009; FAO, 2014) and dewormers to improve the growth and final weight of chickens (Phiri et al., 2007; Chota et al., 2010; Mwale and Masika, 2015; Bessell et al., 2018).

Accordingly, if undertaken alongside ND vaccination, poultry husbandry improvements could lead to a substantial improvement in production and previous studies have shown positive economic returns on ND vaccination, supplementary feeding, housing and parasite control (Jugessur et al., 2006; Njue et al., 2006), but in certain instances there could be a net economic loss (Udo et al., 2006). Therefore, the objective of the study was to add further resolution to the relative contribution of ND vaccination and poultry husbandry in improving poultry productivity and to identify the relative contribution of each practice on impacting smallholder productivity.

## 2 Materials and methods

### 2.1 Survey background

The study was designed to interview smallholder farmers that fall into two categories: 1.) those that adopt ND vaccines and 2.) those that do not adopt ND vaccines. An ND vaccine adopter was defined as any smallholder that answers “yes” to the question “Have you vaccinated your poultry against Newcastle disease in the past 12 months?”. This category includes both smallholders that vaccinate frequently – 3 or 4 times per year which is recommended to ensure protection (Alders et al., 2002) and those that vaccinate less frequently. Hence, we do further analysis breaking down the question “How frequently do you vaccinate your poultry against Newcastle Disease?”. Participants were recruited through a single field survey with an aim of recruiting 50 % ND vaccine adopters and 50 % non-adopters. In order to efficiently identify ND vaccine adopters, the adopters were identified through interviews with agrovet store owners or known village vaccinators (village vaccinators (Bessell et al., 2017) are local individuals that are trained in the storage, preparation and administration of I-2 ND vaccine by eye drop (Alders et al., 2002)). Non-vaccinating households were selected based on convenience sampling from the village population assisted by locally recruited guides who knew the geography of the area. Wherever possible the enumerators maintained a gap of 500 m or 5 houses was between interviewees and the dispersal of interview locations was verified by mapping the interview locations. For the purposes of this study smallholders keeping at least one and no more than 75 chickens were enrolled. The ceiling of 75 was set to ensure that we interviewed smaller scale producers rather than semi-intensive producers operating formal broiler and layer systems. Small extensive and extensive scavenging systems have been classified as farmers rearing 1–5 and 5–50 adult chickens (FAO, 2014). In this study by setting a ceiling of 75 total chickens we are consistent with this classification. This ceiling also ensured that flock size did not become an overwhelming determinant of behaviour.

### 2.2 Questionnaire

The questionnaire was implemented on Android OS smartphones using the Open Data Kit (ODK) App (Hartung et al., 2010). It covers a number of aspects of chicken rearing:

1 Respondent eligibility
2 Basic information – time, date, village, coordinates
3 Background details of the respondent
4 Breakdown of chicken flock
5 Breakdown of chickens gifted and purchased
6 Chicken health
7 Selling chickens
8 Chicken meat consumption
9 Gifting of chickens
10 Egg production, consumption and sales
11 Reasons for death of adult chickens
12 Reasons for death of chicks and growers (defined as juvenile chickens)
13 Uses of revenues from chicken sales
14 Mammalian species farmed

The questionnaire was addressed to the person responsible for making decisions with respect to the chickens. Where it was not possible for one individual in the household to answer all questions, the input of others was sought. The questionnaire survey was coded using xlsForm and the survey is in supplementary information S1 and can be viewed at https://ee.kobotoolbox.org/preview/::942WfITH, note that questions are dependant, so the online survey only opens up after initial questions have responses).

### 2.3 Sample size

Sample sizes were estimated on a per district level as a population proportion based on 50 % sample proportion, 95 % confidence level and a precision of 10 %. Fitting to a population of 35,000 households gave a required sample size of 96 households in each district (Daniel, 1999) and was implemented as a minimum of 50 adopters and 50 non-adopters in each district. However, in practice it was possible to get nearer to 60 adopters and 60 non-adopters in each district further powering the study.

### 2.4 Survey implementation

The questionnaire was written in English and interviews were conducted in English or the local language that the respondents were most comfortable speaking. The interview was conducted in a conversational manner with respondents sometimes seeking animal health advice or asking questions to the enumerators. There was no formal translation to local language(s), instead enumerators were translating as necessary during the interviews and enumerators and respondents sometimes switched between languages during an interview as they felt appropriate or comfortable. Enumerators reviewed and made locally relevant changes to the questionnaires during a training where they pre-tested and practiced the questions until they were fully familiar with the interview. The interview was further pre-tested with selected local smallholders. The study was implemented in September and October 2017.

The research was carried out with the approval by the District Veterinary Offices of the study districts and every district assigned a veterinary officer to work with the research team during data collection within their districts. In accordance with the standards of Uganda, no formal ethical approval was required for this study. Study participants were informed of the purpose of the study and only participated if they agreed to do so. Smallholders were not offered or given any incentive for participation and smallholders’ participation did not impact in any way on future provision or access to services.

### 2.5 Study areas

**Fig. 1:**
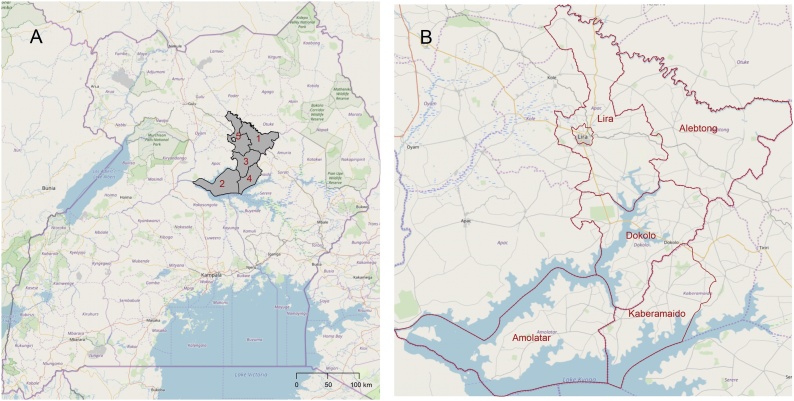
The study areas in Uganda (A) with the districts of Alebtong (1), Amolatar (2), Dokolo (3), Kaberamaido (4), Lira (5). These are zoomed in map B. The basemap is from Open Street Maps (Open Street Map © OpenStreetMap contributors under a Creative Commons Attribution-ShareAlike 2.0 licence (CC-BY-SA)). District boundaries are adapted from GADM (www.gadm.org).

The study was implemented in five districts in Uganda (Fig. 1). The districts were selected as they have similar socio-economic, agro-ecological and ethnic characteristics and were districts where co-authors have been active in setting up ND vaccine distribution networks. Multiple districts were required to control for any district level measures that were taken to control disease spread and district was tested as a co-variate in statistical models. Equal numbers of ND vaccine adopters and non-adopters were targeted for recruitment in each study area. The study targeted 600 smallholders in total (50 % adopters and 50 % non-adopters).

### 2.6 Modelling

Productivity is analysed as a statistical model of the offtake of chickens and of eggs. Chicken offtake is defined as the number sold during the previous 12 months, the number gifted during the previous 12 months and 2 times the number consumed during the past 6 months (data were not collected on consumption over a 12 month period). By incorporating sales over a 12 month period we allow for annual fluctuations in market values that coincide with seasonal variations and market responses to religious festivals.

Different generalised linear model (GLM) frameworks were tested including binomial and Poisson error structures and these were found to be poor fits with large over dispersion. The most appropriate fit was with a negative binomial GLM implemented in R (R Core Team, 2017) using the glm.nb function from the MASS package (Venables and Ripley, 2002).

The model was fitted in a number of stages:

• The offtake is likely to be some function of flock size, or the number of productive chickens in the flock. However, there are a number of ways that flock size could be measured so we tested we fit 5 separate models:

$$\left(model\;1\right)\;offtake\approx a+1+\varepsilon$$

$$\left(model\;2\right)\;offtake\approx a+total\;poultry+\varepsilon$$

$$\left(model\;3\right)\;offtake\approx a+adult\;poultry+\varepsilon$$

$$\left(model\;4\right)\;offtake\approx a+growers+\;adult\;poultry+\varepsilon$$

$$\left(model\;5\right)\;offtake\approx a+chicks+growers+adult\;poultry+\varepsilon$$

Where *a* is the intercept and ε is the error term, the Akaike Information Criterion (AIC) of the 5 models were compared and that with the lowest AIC selected and taken forward.

• The model with the lowest AIC above was fit with the district of the flock. AICs of the models with and without district were tested and the model with the lowest AIC retained.
• Four husbandry factors were tested in turn: use of ND vaccines, ownership of a poultry house for overnight housing, use of supplementary feeding and use of dewormers. Adopters of ND vaccines are defined as those that responded “yes” to the question “Have you vaccinated your poultry against Newcastle disease in the past 12 months?”. The husbandry variables were included and subsequently the least statistically significant was excluded until only statistically significant husbandry factors remain.
• Interactions between the flock size variable(s) and the husbandry practices were tested and retained if they reduced AIC.

The fit of the models was evaluated by inspecting the dispersion and plotting the model residuals. Analysis of diagnostic plots showed one data point that corresponded to a flock of 13 chickens that had an offtake of 134, this was subsequently removed. We had no *a priori* reason to assume that there was spatial structure to the data, but the variogram of the final model’s residuals was inspected for evidence of spatial autocorrelation and there was none.

A similar model was constructed to describe the offtake of eggs during the previous week. Here offtake was described by the sale and consumption of eggs combined. The model was constructed in the same way as the chicken offtake model, but there were no outliers that were identified and subsequently all data were retained.

Chicken sale values were modelled based on the median, maximum and minimum sale values cited by respondents. We fit a triangular distribution using the triangle package in R (Carnell, 2019) to the values for each respondent and drew at least 2,000,000 samples in total from the realm of distributions, sampling from each distribution proportional to the size of that flock.

## 3 Results

### 3.1 Survey breakdown

Rather than the target of 50 % ND vaccine adopters, 52.3 % of respondents were vaccinating against ND and the majority of vaccine adopters vaccinate three times per year (Table 1). More adopters than non-adopters used dewormers on their chickens, and owned a poultry house (Table 1). Most chickens were fed by scavenging alone (Table 1).

**Table 1:** Descriptive statistics to compare the adopters and the non-adopters. The percentages represent the percentage that are in that group (except for the “Overall” row).

	Non-adopters	Adopters
Overall	283 (47.7)	310 (52.3)
Respondent gender:		
Female	166 (58.7)	172 (55.5)
Male	117 (41.3)	138 (44.5)
District:		
Alebtong	53 (18.7)	62 (20.0)
Amolatar	55 (19.4)	61 (19.7)
Dokolo	59 (20.8)	67 (21.6)
Kaberamaido	56 (19.8)	59 (19.0)
Lira	60 (21.2)	61 (19.7)
Frequency of ND vaccination per year:		
Never	283 (100)	0
Once	0	14 (4.5)
Twice	0	66 (21.3)
Three or more	0	217 (70.0)
When vaccinator visits	0	13 (4.2)
Dewormers	23 (8.1)	179 (57.7)
Poultry housing	133 (47)	186 (60)
Feeding:		
Scavenging	238 (84.1)	185 (59.7)
Leftovers	16 (5.7)	23 (7.4)
Poultry feed	29 (10.2)	102 (32.9)

**Fig. 2:**
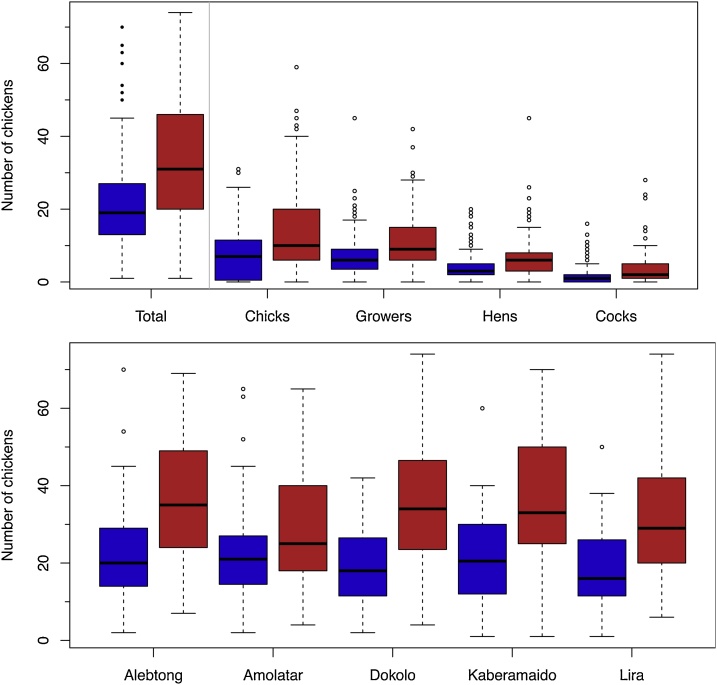
Boxplots of the breakdown of flock sizes by chicken age group and by district. The blue boxes are non-adopters and red boxes the adopters. The boxes represent the interquartile range, the middle lines the medians and the whiskers are 1.5 times the interquartile range, or the limits of the data.

Flock sizes were larger among adopters compared to non-adopters and this difference was consistent across study province or district (Fig. 2).

**Fig. 3:**
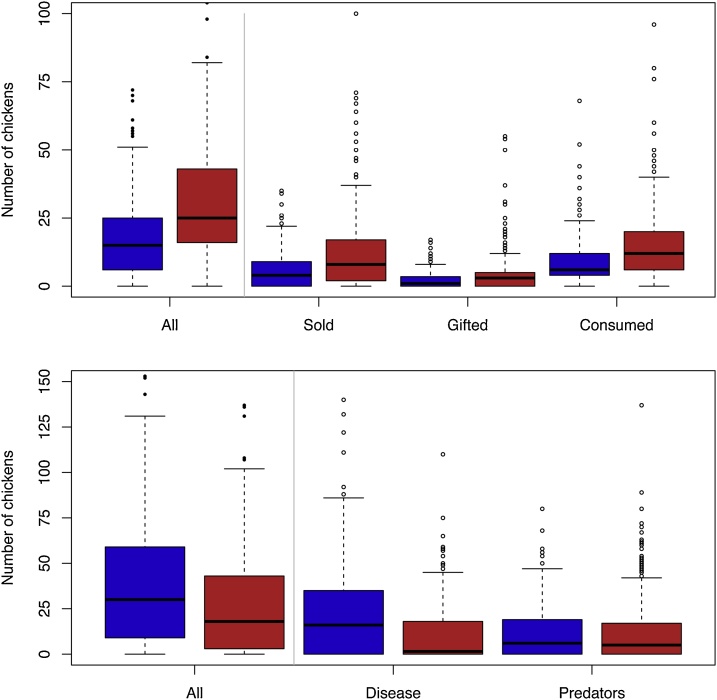
Boxplots of the breakdown offtake (top) and deaths (bottom), the blue boxes are non-adopters and red boxes the adopters. The boxes represent the inter quartile range, the middle lines the medians and the whiskers are 1.5 times the interquartile range, or the limits of the data.

**Fig. 4:**
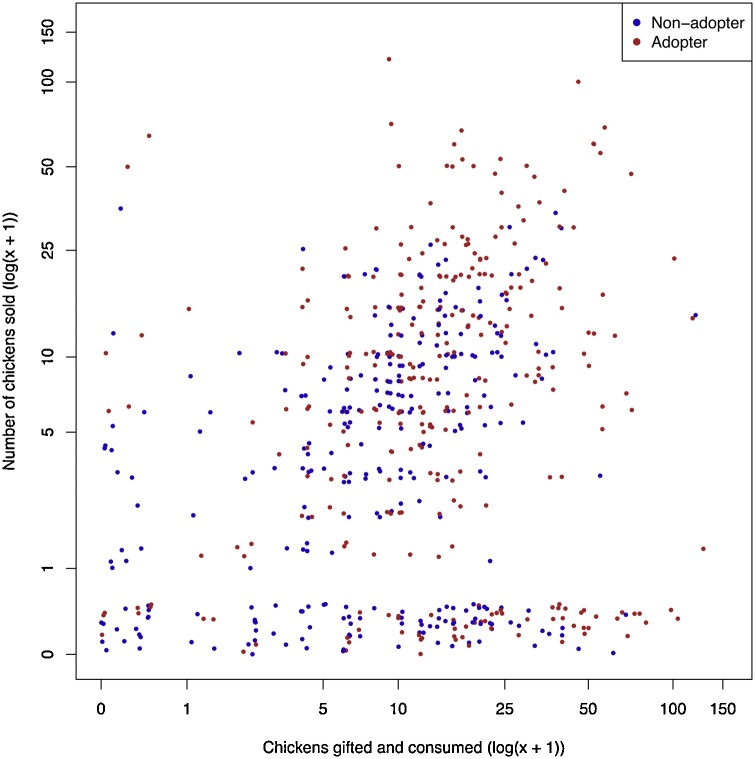
Scatterplot of the breakdown of offtake comparing gifting and consumption of chickens against sales during one year. Points have been adjusted by adding a sampled jitter in the range of 0 – 0.5 to each data point in each axis.

Offtake was greater among the adopters compared to the non-adopters and sales accounted for the greatest proportion of offtake (Fig. 3). There was a greater proportion of deaths due to diseases compared to deaths due to predators among non-adopters, but not among adopters (Fig. 3). Offtake comprises home consumption (plus gifts were included here) and sales and these two metrics are broadly correlated (Fig. 4). However, a large number of respondents reported home consumption but no sales (n = 136), but few (n = 21) had sales but no home consumption.

### 3.2 Economics

**Fig. 5:**
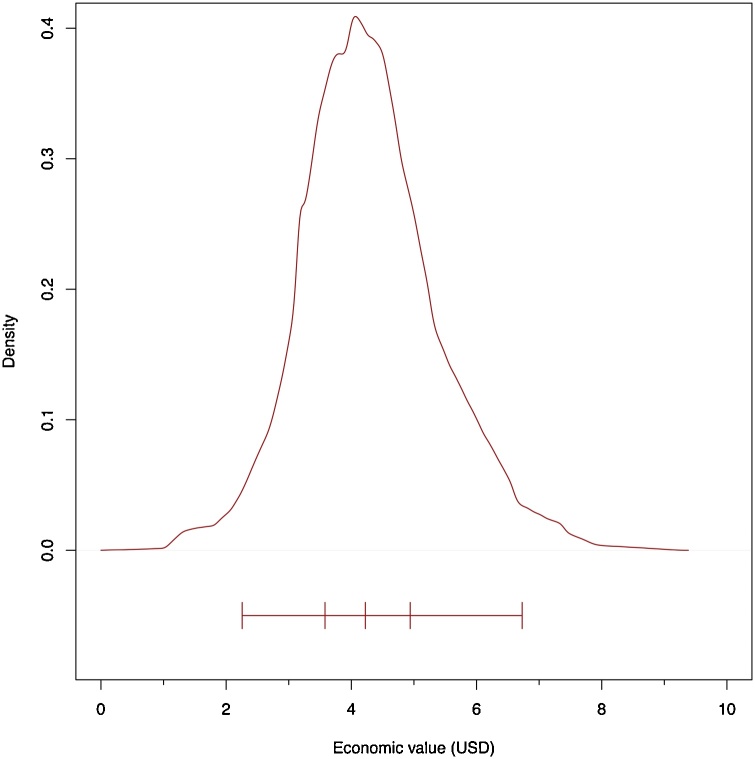
Distribution of sampled values of chicken sale values that were cited by respondents. The top line represents the results of the density function in R using a gaussian kernel. The lower line represents the extent of 95 % of the data, the 25th and 75th percentiles and 50th percentiles.

Modelling the sale values cited by respondents, the median sale value was US $4.22 per chicken sold and there was quite a tight distribution around these sale values (Fig. 5). The median cost per egg sold was 9USc. The median vaccine cost was 3USc per dose per bird.

### 3.3 Modelling

The model with total chickens as the denominator had the lowest AIC, and AIC was further reduced by including district (Table 2). The model with the lowest AIC included ND vaccine adoption and ownership of a poultry house (Table 2).

**Table 2:** Results of the multivariable GLM of offtake with negative binomial error structure. The residual deviance is 678 on 584 degrees of freedom and the AIC 4910.6.

Predictor	Estimate	Std. error	IRR^a^ (95% Cis)	z-value	Pr (> |z|)
Intercept	2.517	0.097	12.39 (10.24–15.05)	25.82	<0.001
District					
Alebtong	0 (Ref)	–	1	–	–
Amolatar	−0.004	0.106	0.996 (0.808 – 1.228)	−0.04	0.968
Dokolo	0.007	0.104	1.007 (0.821 – 1.236)	0.071	0.943
Kaberamaido	−0.312	0.106	0.731 (0.594 – 0.901)	−2.939	0.003
Lira	−0.00004	0.104	1.000 (0.815 – 1.226)	0	1.000
Total chickens	0.011	0.002	1.011 (1.007–1.015)	4.872	<0.001
ND vaccine adoption					
Non-adopter	0 (Ref)	–	1	–	–
Adopter	0.451	0.072	1.571 (1.365–1.808)	6.251	<0.001
Poultry housing					
Not present	0 (Ref)	–	1	–	–
Present	0.311	0.069	1.365 (1.193–1.56)	4.538	<0.001

Ref = Reference level.

Offtake = the number of chickens sold during the previous 12 months, the number gifted during the previous 12 months and 2 times the number consumed during the past 6 months.

a IRR = incidence rate ratio.

Sensitivity analysis including just those households that vaccinate at least three times per year as adopters made no significant change to the model results presented in Table 2.

**Fig. 6:**
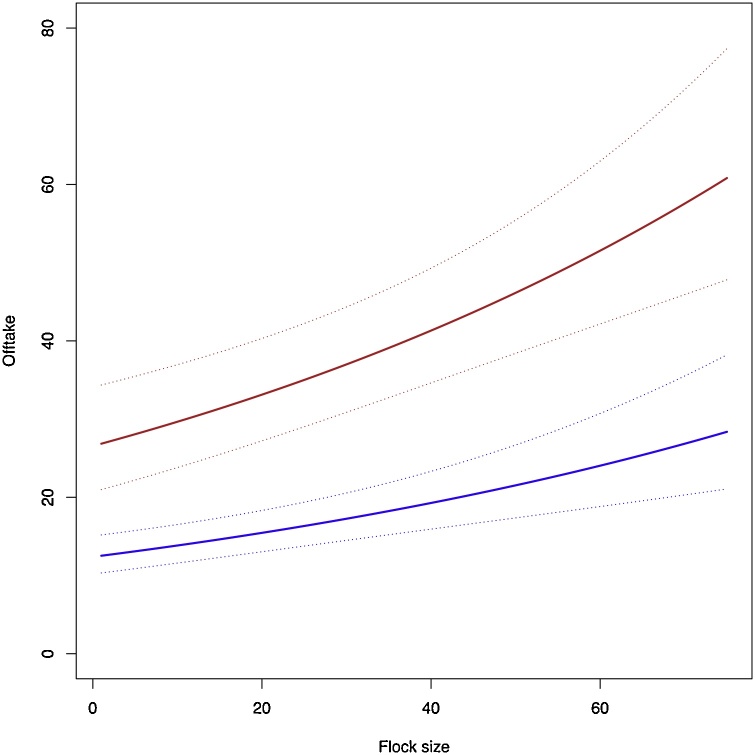
Fitted values for the model for flocks that are non-adopters and do not own a poultry house (blue line), and those that are adopters and own a poultry house (red line), this fitted for Alebtong district. Broken lines represent 95 % confidence intervals.

Plotting the values of predictions extrapolated from the model shows the relative change in offtake with increasing flock size for flocks that have poultry housing and for those that do not (Fig. 6, Table 3).

**Table 3:** Summary of the modelled annual offtake at different flock sizes and for husbandry practices.

	Predicted annual offtake			
Flock size	No vaccine, no poultry housing	Vaccine, no poultry housing	No vaccine, poultry housing	Vaccine & poultry housing
10	13.8	21.7	18.9	29.7
20	15.5	24.3	21.1	33.1
30	17.3	27.1	23.6	37.0
40	19.3	30.3	26.3	41.3

Offtake = the number of chickens sold during the previous 12 months, the number gifted during the previous 12 months and 2 times the number consumed during the past 6 months.

In the model of egg production, a model fitted with the number of hens as the demographic predictor has the lowest AIC, but including district did not reduce AIC. Subsequently, the model with ND vaccine adoption and poultry housing had the lowest AIC, but poultry housing was marginally non-significant and so was dropped from the final model (Table 4). It must be noted that 43.5 % of respondents reported no egg production during the reference time period comprising the previous week.

**Table 4:** Results of the multivariable GLM of egg offtake with negative binomial error structure. The residual deviance is 589.5 on 590 degrees of freedom and the AIC 2742.5.

Predictor	Estimate	Std. error	IRR^a^ (95% Cis)	z-value	Pr (> |z|)
Intercept	0.545	0.132	1.725 (1.324–2.264)	4.137	<0.001
Number of hens	0.090	0.017	1.094 (1.056–1.136)	5.192	<0.001
ND vaccine adoption					
Non-adopter	0 (Ref)	–	1	–	–
Adopter	0.588	0.150	1.801 (1.343–2.412)	3.921	<0.001

Ref = Reference level.

Offtake = The number of eggs sold and consumed during the week before the interview.

a IRR = incidence rate ratio.

## 4 Discussion

We have collected data and parameterised a model to describe the impacts of different husbandry practices on the level of offtake from smallholder chicken flocks in Uganda. Ideally, this type of study would require long term longitudinal monitoring of the study flocks to record the inputs, outputs, births and deaths, sales and consumption. However, a study of this nature represents a considerable logistical challenge on even a relatively small scale (Henning et al., 2008). Hence, in order to conduct these analyses over large geographical areas, we used a cross-sectional study that considered smallholders’ activities over the previous year and fitted the model to account for the likely shortcomings in the data collection framework. Consequently, the study relies on the recollection of the farmers of their activities over the previous year and could be prone to recall bias. Whilst it would be beneficial to use farmer record books, many smallholder farmers do not keep records of their activities so record books can not be used to give a representative sample of smallholders.

One of the principal drivers of productivity will be the number of chickens in the flock and in particular the number of productive chickens. The number of new chickens entering the flock over a period of time will generally be a function of the number of productive hens during that time, a number which will change during the time period. However, due to the cross-sectional nature of the data collection in this study, we do not have a reliable estimate for the number of hen days, so we tested four different proxy variables, considering: 1) the total number of chickens, 2) only the number of adult chickens, 3) the adult chickens and growers separately and 4) the adults, growers and chicks separately. In this model, the best predictor was the total number of chickens, indicating that the wider flock dynamics needed to be included beyond just the number of adult chickens. However, flock size was a predictor of offtake but not a particularly strong predictor and this is in part due to the flock size being recorded at the end of the period of offtake and so it does not account for events such as die-offs, or decisions to sell or consume productive chickens. That a static measure of the flock’s size is not a good predictor or productivity indicates that there are a lot of other potential drivers of production that could be considered here.

The differences in management practices are emphasised by Fig. 4 which shows there are a large number of flocks that can be very productive but rear chickens purely for home consumption or for gifts and do not sell chickens. This potentially produces a very different dynamic with chickens likely consumed in small numbers (one or two chickens) at regular intervals whilst with sales we might expect the smallholders to sell multiple chickens at irregular intervals. The model was run separately with the outcome as either sales or gifts + consumption and the same predictors remained significant. However, due to the zero-inflation problem, the sale model was a poor fit and so is not presented.

ND vaccination was identified as a significant contributor to flock productivity with an increase in flock chicken productivity of 57 % after other management practices that affect flock productivity had been taken into account, which is generally greater than increases in off-take observed elsewhere in Africa (Fisher, 2014). This compares to returns on investment of ND vaccination using the F strain by nose drop administration in Kenya of 3.36 and of 1.15 from supplementary feeding for 16 farms in Kenya (Njue et al., 2006). A similar small scale study in Mauritius found similar returns on investment from ND vaccination using live NDV4 vaccine by eye drop or drinking water of 4.2–6 (Jugessur et al., 2006). The corresponding impact on egg production was greater still at 80 % which whilst the value of eggs is smaller than chickens, eggs represent an important source of regular income and protein (Sonaiya and Swan, 2004).

In this study in Uganda it is conspicuous that the majority (70 %) of respondents were compliant with the recommended cycle of vaccinating 3–4 times per year (Alders et al., 2002) (Table 1), of those remaining, 21.3 % vaccinate twice per year. Accordingly, sensitivity analysis including only adopters that vaccinate at least three times per year made no significant difference to the predictors. This compliance to the vaccination regime may contribute to the magnitude of the impact of ND vaccination.

It should be noted that the beneficial impact of productivity among ND vaccine adopters was not observed in similar studies that were conducted in Burkina Faso and India. In Burkina Faso, there was a positive benefit with adopters 17 % more productive, but once the costs of the inactivated ND vaccine are considered the net economic benefits are marginal (unpublished data). As we did not collect disease data from study flocks we do not have the data to explain this result without resorting to pure speculation and so we have not presented the results. In India no effect was seen in a much smaller and geographically limited study (unpublished data).

For the model of flock chicken production, the ownership of a poultry house had a significant effect, but not deworming or supplementary feeding. The non-significance of dewormers could be due to wider management practices such as rereleasing chickens to contaminated environments or because we measured the absolute number of chickens taken off, rather than the weight of those chickens, and indeed the impact of dewormers on chicken weight is highly variable (Phiri et al., 2007; Chota et al., 2010; Mwale and Masika, 2015; Bessell et al., 2018). Similarly for supplementary feeding, the condition of the chicken was not considered in this study, purely productivity measured by numbers of chickens.

Whilst these analyses focussed on four facets of poultry production – vaccination, deworming, feeding and housing and that there are a number of additional factors that are not considered here. In the outcome we consider only offtake of chickens and eggs. However, the objectives of chicken production may be different and in particular in the context of this study smallholders may be seeking to hold on to birds in order to grow the flock. A further constraint on productivity is the lack of effective marketing channels for rural poultry, marketing, market instability and supply fluctuations often acts as a considerable constraint on making a profit from selling poultry produce (Sonaiya and Swan, 2004; Queenan et al., 2016)

A limitation on this study was the necessary ceiling of 75 chickens that was placed on the size of the flock. This places something of an artificial constraint as the better, more productive flocks may have grown beyond this level. It is noticeable in Fig. 2 that the sizes of adopter’s flocks are pushing the upper limit on flock size. This ceiling on flock size may have an impact of reducing the observed impact of productivity by excluding those flocks that have seen the greatest increase in production.

Whilst the enumerators report that almost all farmers that were approached agreed to participate in the study, we did not record the numbers of farmers that declined to participate. In future studies this would be a valuable addition to the study design as the numbers that refuse to participate gives an estimate of the unobservable population. Furthermore, we did not record any interviews for data quality verification. Whilst this would be valuable, the recording could change the dynamics of the interview, or lead to farmers declining to participate.

In the study population there was no significant impact on the gender of respondent on production, and the gender of the respondent was no significant difference between genders in terms of ND vaccine adoption. However, poultry production is know to be strongly gendered in nature and in other settings gender is known to influence ND vaccine adoption (Alders et al., 2010; Campbell et al., 2018). Future studies of this nature would benefit from taking sociocultural issues and the role of women in poultry rearing and could be structured to ensure that different households structures are represented in the sample and within this consider the sometimes complicated decision making structures that exist (Guèye, 2000).

Due to the challenges of field logistics, we did not record any breakdown of the different input costs to construct a full economic model. To do this would require a different study design involving a longitudinal study of a small number of households (Henning et al., 2008, 2009, 2013), rather than a large cross-sectional survey implemented here. Hence the model that we have developed is a model of the number of offtakes and from the modelled value data, we can infer the value of this production.

Whilst further economic analyses are required to understand the wider economic benefits of husbandry practices, the input costs are relatively low. The cost of 3USc per vaccine dose per chicken, amounts to 2.40 USD to maintain vaccination for one year in a flock of 20 chickens on a 3 month vaccination regime. This compares favourably to $41.36 gross benefits in meat production (if we assume that the value of a chicken that is gifted or consumed is equal to one that is sold) and an improvement in egg production amounting to $2.81 per year from a flock with 5 hens. This represents clear benefits to the smallholder as well as additional benefits in terms of the greater certainty that the flock will survive an ND outbreak.

## 5 Conclusions

We have demonstrated that in this study area that ND vaccination is a key intervention that can have a substantial effect on chicken flock production measured by sales, consumption and gifting of chickens and eggs. Whilst housing also impacts on chicken production, ND vaccination had the biggest effect. This is consistent with results from elsewhere, and enforces the need for vaccination against ND and key diseases for improving livelihoods of rural communities.
